# Endoscopic removal of magnetic beads causing duodenal chronic fistula

**DOI:** 10.1055/a-2358-1405

**Published:** 2024-07-29

**Authors:** Jingjing Yao, Jindong Fu

**Affiliations:** 1 Department of Gastroenterology, Rizhao People’s Hospital, Rizhao, China

A 4-year-old girl with pituitary dysplasia for 2 years presented to our hospital after swallowing a coin 3 hours earlier. An abdominal X-ray revealed the coin in her stomach, along with an unexpected string of beaded, high-density images in the right upper abdomen ( Fig. 1 ). Her parents disclosed that the girl had played with magnetic beads a month prior and might have ingested them then, but she had no symptoms of abdominal pain, vomiting, or fever. Under intravenous anesthesia, the girl underwent gastroscopy.

**Fig. 1:**
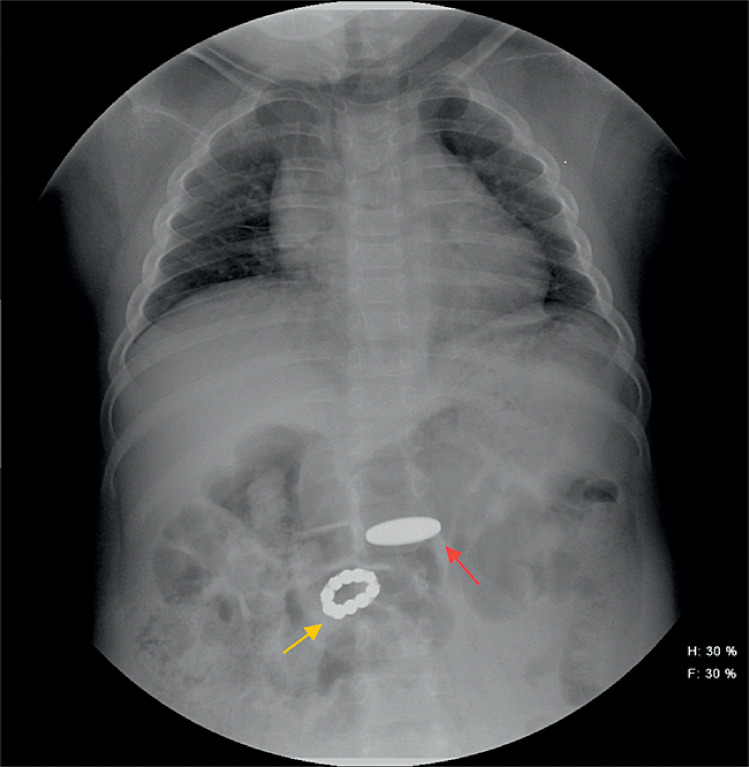
Abdominal X-ray showed the image of the coin in the stomach (red arrow), and a string of beaded, high-density images, forming a ring, in the right upper abdomen (yellow arrow).

A coin was identified in the stomach ( Fig. 2 ) and removed using a foreign body forceps. Additionally, a string of magnetic beads with corroded surfaces was discovered in the descending duodenum, with one end protruding from the intestinal cavity, while the other end was invisible ( Fig. 3 ). Given that it had been a month since ingestion and the X-ray showed the beads forming a ring, we inferred that a chronic fistula might had developed due to the magnetic force of the beads. Attempting endoscopic removal, we grasped one bead firmly with the forceps and successfully removed all the beads with the aid of a transparent cap ( Video 1 , Fig. 4 ). After removal, a fistula was exposed with no bleeding and there was no damage to the intestinal mucosa ( Fig. 5 ). A nasointestinal tube was used to maintain nutritional intake. Postoperatively, no complication occurred. Abdominal X-ray also revealed no complications. The girl was discharged 2 days later.

**Fig. 2:**
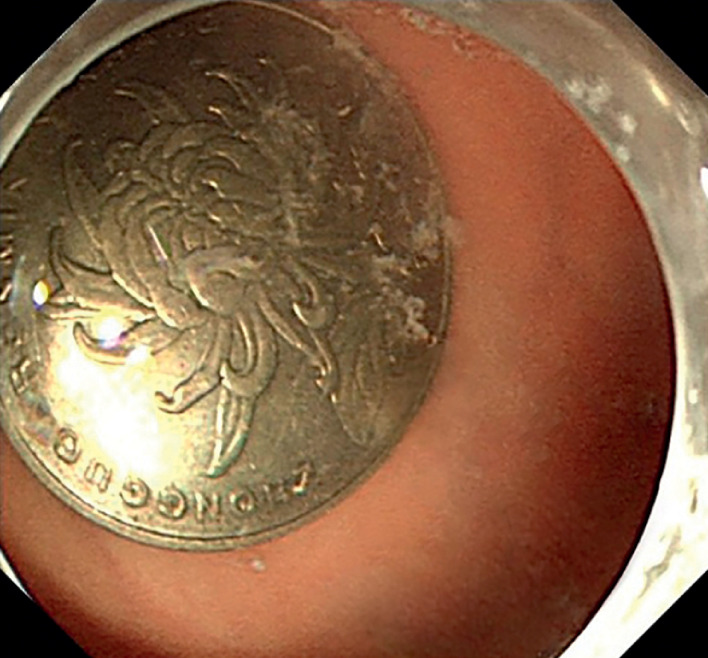
Endoscopy showed a coin in the stomach cavity.

**Fig. 3:**
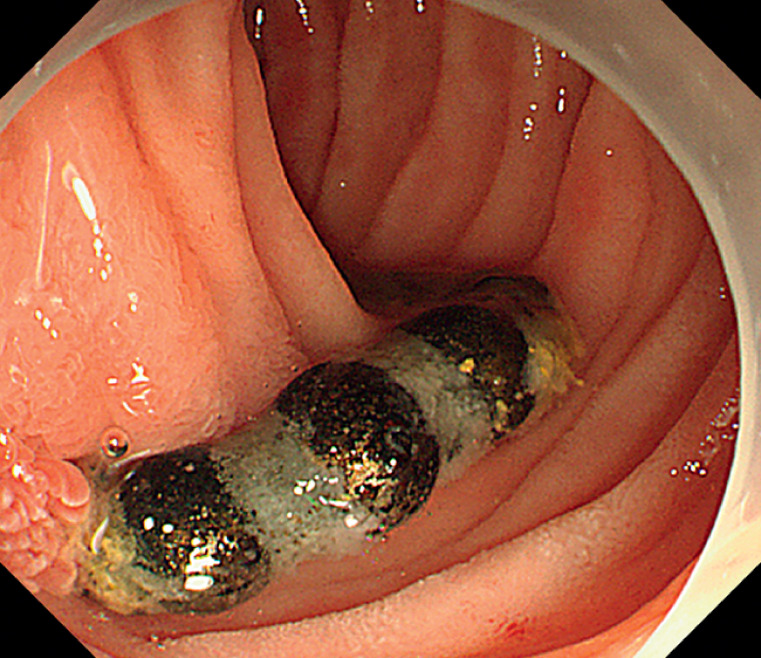
A string of magnetic beads with corroded surfaces was discovered in the descending duodenum, with one end protruding from the intestinal cavity.

Endoscopic removal of the magnetic beads.Video 1

**Fig. 4:**
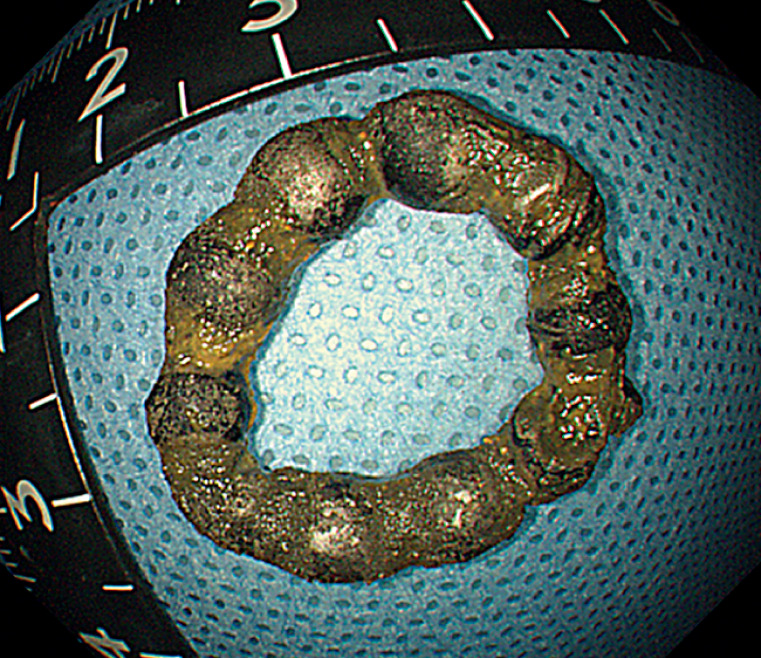
The removed magnetic beads.

**Fig. 5:**
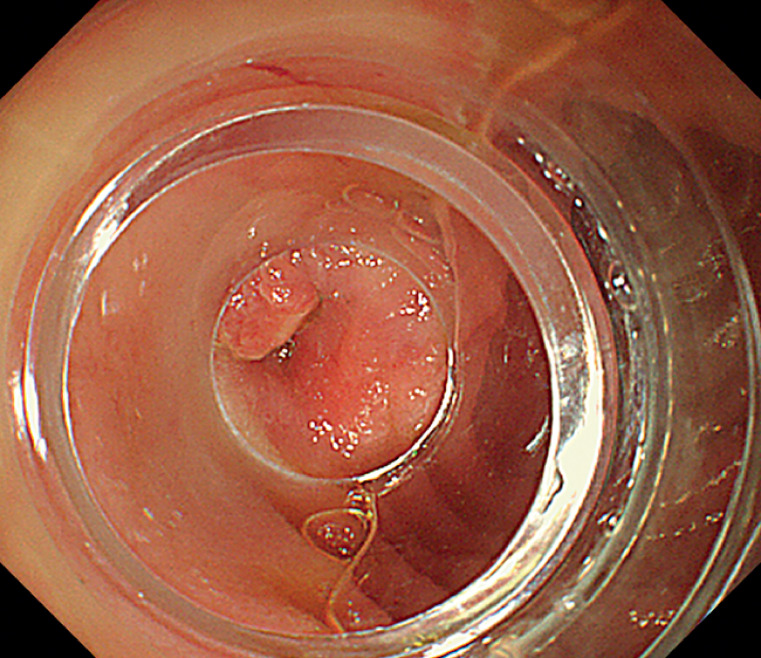
A fistula was exposed with no bleeding.

The incidence of injury from magnetic beads in pediatric patients has increased in recent years. When more than two magnetic beads are ingested, the intestinal walls are tightly attracted to each other, leading to necrosis and fistula formation 1 , often requiring surgical intervention 2 . In this case, the swallowed beads were discovered relatively late due to the absence of symptoms, and a chronic fistula had developed. Our experience suggests that endoscopic removal of magnetic beads can be safe in the presence of chronic fistula.

Endoscopy_UCTN_Code_TTT_1AO_2AL

## Notes

Endoscopy E-Videos https://eref.thieme.de/e-videos
*E-Videos* is an open access online section of the journal *Endoscopy* , reporting on interesting cases and new techniques in gastroenterological endoscopy. All papers include a high-quality video and are published with a Creative Commons CC-BY license. Endoscopy E-Videos qualify for HINARI discounts and waivers and eligibility is automatically checked during the submission process. We grant 100% waivers to articles whose corresponding authors are based in Group A countries and 50% waivers to those who are based in Group B countries as classified by Research4Life (see: https://www.research4life.org/access/eligibility/ ). This section has its own submission website at https://mc.manuscriptcentral.com/e-videos .
